# SEFRE: Semiexoskeleton Rehabilitation System

**DOI:** 10.1155/2016/8306765

**Published:** 2016-08-04

**Authors:** Winai Chonnaparamutt, Witsarut Supsi

**Affiliations:** National Electronics and Computer Technology Center, 112 Thailand Science Park, Pathum Thani 12120, Thailand

## Abstract

SEFRE (Shoulder-Elbow-Forearm Robotics Economic) rehabilitation system is presented in this paper. SEFRE Rehab System is composed of a robotic manipulator and an exoskeleton, so-called Forearm Supportive Mechanism (FSM). The controller of the system is developed as the Master PC consisting of five modules, that is, Intelligent Control (IC), Patient Communication (PC), Training with Game (TG), Progress Monitoring (PM), and Patient Supervision (PS). These modules support a patient to exercise with SEFRE in six modes, that is, Passive, Passive Stretching, Passive Guiding, Initiating Active, Active Assisted, and Active Resisted. To validate the advantages of the system, the preclinical trial was carried out at a national rehabilitation center. Here, the implement of the system and the preclinical results are presented as the verifications of SEFRE.

## 1. Introduction

Aging era is now. Based on Thai Aging Status Report, now the elders are around 12% of Thai population, and the percentage can be double in year 2030 [1]. More elderly require more caretakers to support their declined physical abilities, for example, low vision, hearing problem, and weakened muscles. Regarding these physical impairments, there is not only dysfunction from the aging phenomena, but also the disability that is caused by chronic diseases or an accident which must be concerned. In most cases, unusable limbs might be a result for all.

An impaired ability plagues their daily life. Thus relieving any of those impairments is always a great help for them. In general, recovering functions of limbs are practicable. Therefore we focus our research on the rehabilitation of arm and leg. Since recently there are an inadequate number of caretakers, so we believe that employing robotic systems in the rehabilitation process is a must.

Robotics enhances a simple device to be the super power tool. Extra enrichments include repeatability, high precision, and customizable movement. A number of medical and rehabilitation robotic systems have been on trial, while some of them are accepted in a certain level [2–5].

On one hand, numerous robotic rehabilitation systems have been developed around the globe as some examples are listed in Table 1. Many more existing systems have been also collected by Maciejasz et al. [6]. On the other hand, putting one on the market might be burdensome due to several factors, for example, overlarge size and weight or less benefit-to-cost ratio. Another burden is the complication of utilizing the device by a patient or a caretaker. Also, the cost of the systems and services put them too far to be reached.

Thus a device that is bearable in price and competent in features is needed to expand the deployment of robotics in the rehabilitation process. Setting this as our motto, WEFRE (Wrist-Elbow-Forearm Robotic Economic) Rehab System was firstly developed. This system is aimed at being employed as a household tool [7]. Successively, SEFRE (Shoulder-Elbow-Forearm Robotics Economic) Rehab System has been developed as an innovative machine for providing the rehabilitation service in a hospital [8]. SEFRE is designed to let everybody earn the benefits from the system, for example, a patient who has an impaired arm or a healthy person with a problem of muscle deficiency. In this paper, the development of SEFRE is thoroughly explained in Section 2. Then, Section 3 presents the preclinical trial of the system and is followed by the conclusion in Section 4.

## 2. SEFRE Rehab System

SEFRE Rehab System is created as a robotic rehabilitation system for all and sundry. The system is composed of three key components: a Shoulder-Elbow-Forearm Rehabilitating Mechanism, an Intelligent Controller, and a Friendly Graphic User Interface. To expedite the development process, a small industrial robot (KUKA KR5 sixx850) is used for motioning shoulder joint. Then a novel exoskeleton so-called “Forearm Supportive Mechanism (FSM)” is integrated to the system. FSM is responsible for moving elbow and forearm. This section provides the understanding of SEFRE in the details of FSM, rehabilitation protocol, control scheme and implementation, and games. (Details of control design in this section was presented at ECTI 2013, Thailand [8].)

### 2.1. FSM

While the main task of KUKA is to restore the shoulder motions, FSM is deployed as elbow-forearm trainer. FSM has been created as an independent module that can either work on its own or be controlled by other systems. The control program of FSM thus has been developed separately from the main controller, called “sFSM” (Section 2.4.3). FSM has been designed as a lightweight mechanism equipping with a maximum raising power to motion 4 kg of load. To simplify the system, two on-the-shelf servomotors (Dynamixel EX-106+) were integrated to provide the movements of elbow and forearm.

Since FSM must be attached to the patient arm, several criteria need to be considered. These include, for examples, form and material causing no pain or irritation, weight being light to minimize an additional payload to KUKA, and the mechanism suiting for both left and right arms.

**Figure 1:**
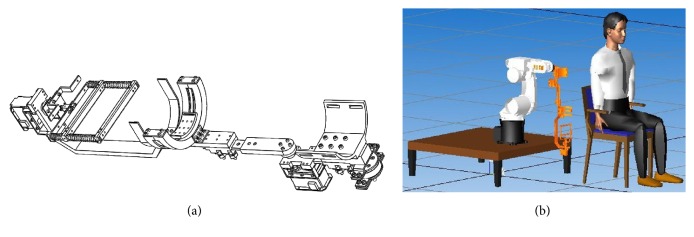
The first prototype of FMS (a) and the configuration of SEFRE Rehab System with a user (b).

As a result, an exoskeleton is a desired form of FSM. This leads SEFRE Rehab System to be a semiexoskeleton robotic rehabilitation system. The first prototype of FSM is shown in Figure 1(a).

After integrating FSM to the robotic manipulator, SEFRE Rehab System is ready to provide an arm therapy to the patient.  Figure 1(b) shows the system configuration when a patient is positioned in front of SEFRE Rehab System.

### 2.2. Rehabilitation Protocol

**Figure 2:**
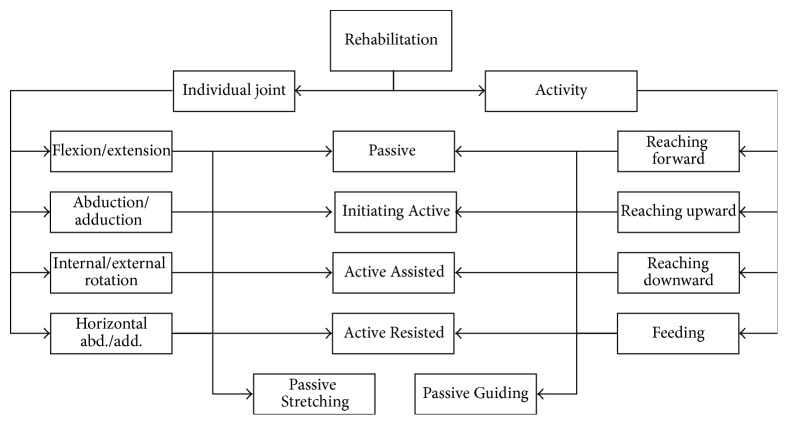
Rehabilitation protocol.

Since we were born, our upper limbs are crucial parts for manipulating things all day and night. Dispossessing the ability to move an arm freely is alike of having no arm. Thus one who loses the limb functions needs to reinstate the features. There are several levels of arm disability based on the residue muscle strength. SEFRE is designed to service the patient in any level of muscle weakness. The system also provides the exercise in two types: the individual joint exercise that let the patient to rehabilitate any dysfunction joint, that is, shoulder, elbow, or forearm, separately one by one, and the combined joints exercise, that is, Functional Activity Rehabilitation, that allows the patient to move the arm in a pattern of an activity in a daily life.  Figure 2 presents the complete rehabilitation protocol of SEFRE Rehab System.

In the individual joint exercise, five therapy modes are provided based on each Muscle Strength Level (MSL).

#### 2.2.1. Passive (P)

Passive mode provides complete support to produce a joint motion of the target joint within the selected range of motion (ROM). The movement is carried out by SEFRE without any effort from the patient.

This mode is used for the patient with MSL 0 who does not have any residue muscle strength, that is, any patient who completely lost the muscle strength by a disease or an accident.

#### 2.2.2. Initiating Active (IA)

In this mode, a joint motion must be initiated by an acting force from the patient; then the motion is carried out by SEFRE as in Passive mode. This is a motivation mode that encourages the patient to try to use the regain muscle force.

This mode is used for the patient with MSL 1-2 who begins to recover some muscle strength. This could be a next step of rehabilitation process after the patient did exercises of the Passive mode in a period of time.

#### 2.2.3. Active Assisted (AA)

The AA mode provides for a patient who can insert a target guiding force to the system in some period of time. After the guiding force is less than the target level, SEFRE continues the motion as in Passive mode.

This mode is used for the patient who recovers and reaches the muscle strength in level 3 who wishes to train oneself to gain more and more strength.

#### 2.2.4. Active Resisted (AR)

This mode is similar to AA except that SEFRE only moves when the guiding force is more than or equal to the target level. This is a weight-training for the patient who almost completely recovers oneself.

This mode is used for the patients with MSL 4-5 who has high level of muscle strength. The patient who practices in this mode has ability to do the daily activities almost similar to a healthy person.

#### 2.2.5. Passive Stretching (PS)

This is a special mode that is available only for the individual joint exercise. In this mode, a joint motion is carried out by SEFRE as in Passive mode. The additional step is a pause for a short period of time at either end of the desired path. This mode let ROM of the joint be increased by stretching the joint at either end.

This mode is used for the patients with MSL 0–5 who has a spastic problem.

#### 2.2.6. Passive Guiding (PG)

This is a special mode that is available only for the functional activity exercise. For the functional activity option, the patient can exercise based on a typical arm movement, for example, reaching forward or feeding oneself. Four first therapy modes are provided the same as in the previous exercise type, that is, P, IA, AA, and AR. And in PG mode, a desired moving path is defined by a doctor or a caretaker, that is, a special reaching pattern; then this new customized path can be added for practicing in Passive mode.

This mode is used for the patient with MSL 0 who does not have any residue muscle strength who requires additional special movement paths.

### 2.3. Control Scheme

**Figure 3:**
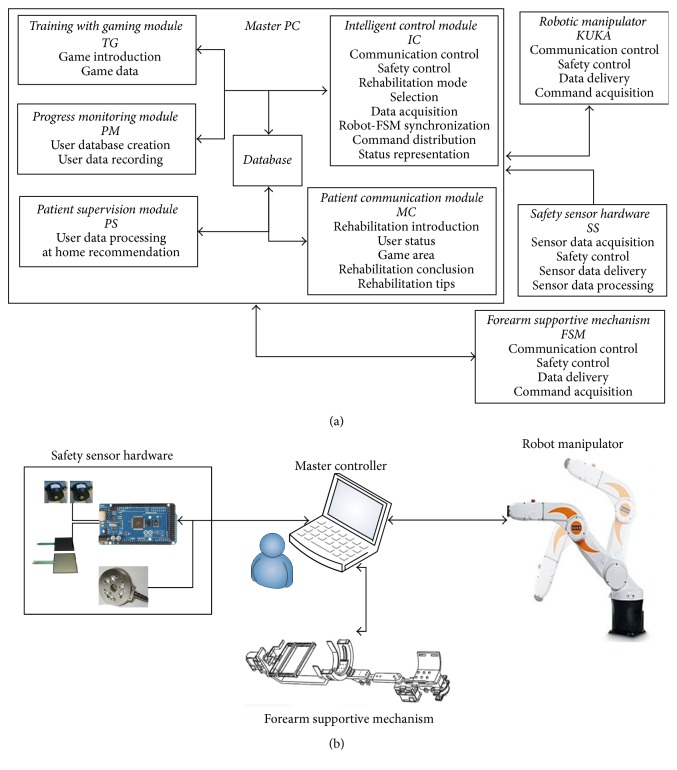
Overview of control system for SEFRE Rehab System: (a) scheme and (b) layout.

SEFRE is targeted as an intelligent device that any caretaker or even patients themselves can use the tool enjoyably with no sweat. A number of key components thus have been evolved: Intelligent Control System providing effective rehabilitation to all, Friendly GUI and Games pleasing and entertaining the patients (Section 2.5), and Database Modules collecting and providing the fruitful data for the doctors and therapists. The overview of control system is shown in Figure 3(a).

The control system is divided into four modules: Master PC, Robot Manipulator (KUKA), Safety Sensor Hardware (SS), and FSM (Figure 3). As defined by its name, Master PC is the primary unit to administer the activities of the rest, especially keeping an eye on SS. SS module is the Interpol of error monitoring units in the system. These include User Ready signal, End-Effector Motion signal, Robot Range Motion signal, Enabling Switch signal, and Emergency Stop signal. Every fault signal is delivered to both the manipulator and Master PC to halt the system till the error is acknowledged, clarified, and solved. To conduct an exercise for the patient based on rehabilitation protocol, the control scheme of the system is divided into high and low level controls. The high level control tasks are managed by Master PC, while the manipulator and FSM operate the low level control. All tasks are carried out after rehabilitating options are fulfilled.

### 2.4. Control System Implementation

The Master PC has been developed as an Intelligent Controller. The controller is decomposed into five submodules: Intelligent Control (IC), Patient Communication (PC), Training with Game (TG), Progress Monitoring (PM), and Patient Supervision (PS). Each has a key task as follows:

 IC: the intelligent control unit,
 PC: the interface unit between the patient and the system,
 TG: the games management unit,
 PM: the rehabilitation monitoring unit,
 PS: the analyzing and supervising unit.

**Figure 4:**
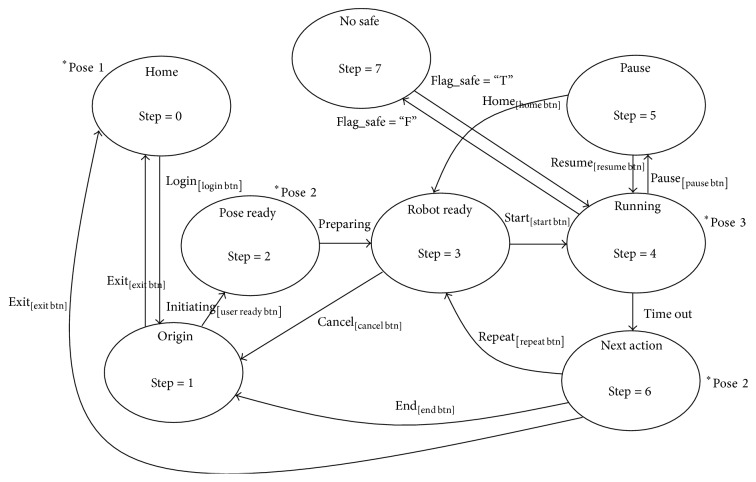
SEFRE state diagram.

IC is responsible for generating commands based on the configured rehabilitation options. To conduct such a task, the communication protocol between IC and other modules, that is, KUKA, FSM, GUI, and Games, is executed as shown in Figure 4.

The protocol has four components: Robot Pose, Robot State, Changing State, and Clicked Button. To change the robot state when a button on the GUI is clicked, IC must send a corresponding signal to the robot after receiving the signal based on the Clicked Button. Consequently, this results in a new pose of the manipulator; that is, Robot Pose and Robot State are changed. This concept opens the door for assembling the other modes to the system by modifying IC only; neither KUKA nor FSM needs to be reprogrammed.

#### 2.4.1. Communication

There are five communication paths to be managed by IC for conducting the rehabilitation process.

*The Manipulator Communication*. This protocol lets IC control the manipulator state as shown in Figure 4. The diagram shows eight major states of SEFRE for conducting the rehabilitation process. For examples, the Home state is the state of KUKA Home position, which allows the manipulator to be safely relocated. Or the Pose Ready state is the state that lets the patient attach the arm to FSM. Or the patient is exercised based on the prior configuration when IC is in the Running state. Therefore, the Running state is a special one that varies the movement of the manipulator. To change the state, transition conditions are controlled by IC exclusively. For instance, after the patient has initiated the rehabilitation via GUI, IC sends an initiating signal to change the robot state from Origin state to Pose Ready state. Then the manipulator adjusts its position to allow the patient to attach the arm properly.

*sFSM Communication*. The communication between IC and FSM controller is similar to the manipulator communication as mentioned above. This allows IC to control KUKA and FSM to change their states simultaneously.

*Force Sensing Communication*. IC communicates with force sensing as essential inputs of the system. Force sensing communication aims to sense force of muscle strength at any joints in Active modes. A variety of force sensing in the system and the purposes of each sensor are deepened in the next topic.

*GUI and Game Communication*. GUI and Game (GG) module is responsible for interacting with the patient in attractive and friendly way. Even though GG seemingly lets the patient give a command directly to the system, the module is not able to understand and execute the patient desire. The information is forwarded to IC for identifying the request and executing the inquiry in an effective way. Also, IC transfers the position of the manipulator back to GG for maneuvering an animation in the game.

*Database Communication*. IC transfers all needed practical data to be recorded in database through Database communication.

#### 2.4.2. Safety Control

IC inspects safety of the system in different patterns based on the state in Figure 4. This task is done by considering signal values from several sensors such as an emergency stop, load cells, or limit switches. For examples, before the rehabilitation begins, IC checks the limit switches whether the patient arm is positioned properly and consistent with the configured side. Or before terminating the rehabilitation process, IC rechecks the limit switches whether the arm has already been detached from FSM. Furthermore, force values from the sensors are determined by IC to judge whether muscle strength exceeding the safety level.

#### 2.4.3. sFSM

sFSM is an autonomous controller to control only FSM module, which is created separately from IC. Two servomotors that are deployed for FSM have two operation modes. Both are joint mode and wheel mode, which are used for controlling motor position and velocity, respectively. Due to mechanical design of FSM, one motor is operated in wheel mode, which requires an additional control algorithm. The algorithm composed of a round-counting function as an encoder and PID (Proportional-Integral-Derivative) control function. This customized algorithm supports sFSM to control the position of the joint while the motor is operated in wheel mode.

#### 2.4.4. Force Sensing

Force Sensing is an essential part of the system because the sensors empower SEFRE to sense any effort from the patient. Each force sensor is selected based on its special properties that are consistent with the sensing task. Three types of force sensors, namely, six-axis force torque sensor, load cell, and Force Sensing Resistor (FSR), are integrated with SEFRE.

*Six-Axis Force Torque Sensor*. A 6-axis force/torque sensor (ATI mini45) is applied for monitoring the motions of shoulder, that is, by sensing the force magnitude and direction exerted by shoulder, which is the most complicated joint of the arm. A high-precision-high-cost sensor is deployed due to the complicated movement of shoulder in six degrees of freedom.

*Load Cell*. Two load cells are applied to measure the force magnitude and direction of elbow: flexion and extension.

*Force Sensing Resistor (FSR)*. Two square FSRs are applied to measure the force magnitude and direction of forearm: supination and pronation.

As a remark, since forearm and elbow rotate around their own axis, so we simplified our system by using load cell and FSR instead of other complicated sensors.

#### 2.4.5. Synchronization

In every state, IC plays a vital role in synchronizing between KUKA and FSM to make various desired motions, for example, reaching forward, to be a concurrent and natural motion. This section informs the synchronization process between both modules of each rehab mode.

**Figure 5:**
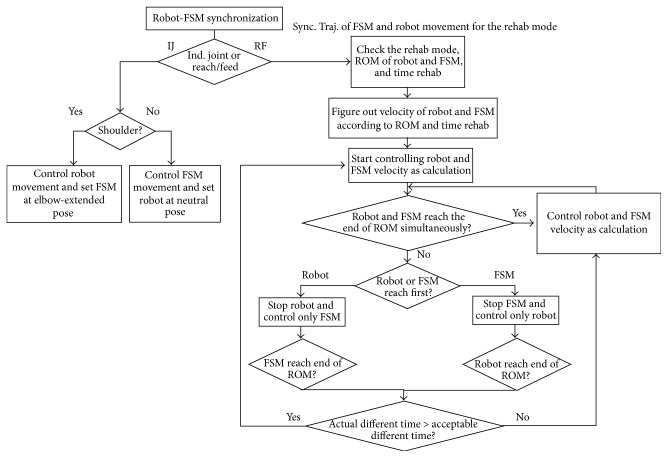
Synchronization procedures between IC, FSM, and KUKA.

According to the synchronization flowchart in Figure 5, IC appoints the velocities of the manipulator and FSM before outsets of their motions concurrently. The tempos are determined based on the exercise speed setting by the patient. Then IC signals to the manipulator and FSM to start moving. In each round, when both are nearly getting back to the beginning point, IC checks whether they reach to the position simultaneously. If so, IC continuously lets them onward, otherwise, the module that has arrived at the point first is paused to wait for the other by IC. After that IC considers the different time interval between two arrival times of KUKA and FSM. The difference is compared with the acceptable time interval, which is 3 seconds based on trial and error. If the different interval is less than or equal to the acceptable interval, IC allows them to move with the current velocities. Otherwise, IC tries to concurrent both modules by adjusting a new velocity for each before starting the next round. The process continues till the end of the session.

Synchronization can be classified based on two major modes: Passive and Active.

*Passive Mode*. IC synchronizes the manipulator and FSM together excluding force sensing communication for arm motions. The force sensing is incorporated only for the safety purpose.

*Active Mode*. IC synchronizes the manipulator and FSM based on the force sensing. So the patient needs to exert force to the system to do an exercise with a preprogrammed path.

### 2.5. Games

To serve various patients who have different conditions of muscle weakness, the exercises are also grouped into two modes: Passive and Active, as explained in the rehabilitation protocol. Therefore, games are designed deliberately to match the patient condition in each mode.

Games nowadays are mostly suitable for Active mode due to they must allow the players to experience the social interactions [22]. This means a player can move actors or objects in the games in any direction any time freely. But this type of game is definitely not suitable for the patient who has no muscle strength. Also, in Passive mode, the robot is moved automatically and disregards any interaction between the system and the patient. Our games thus are studiously designed to be consistent with the movement types and to let the patients enjoy the game in this mode.

#### 2.5.1. Passive Game Design for Rehabilitation

The crucial factors that separate games in Passive mode from the others are the event conditions and scoring.

*Event Conditions Design of Passive Games*. In this game type, the game events occurred only when the joint reaches either end of ROM. For example, to exercise the elbow joint between extension of 0 degrees and flexion of 80 degrees, there is an key object following the elbow motion in the game. So an event in the game only happened if the object is located nearly or exactly at either the position of extension of 0 degrees or flexion of 80 degrees.

**Figure 6:**
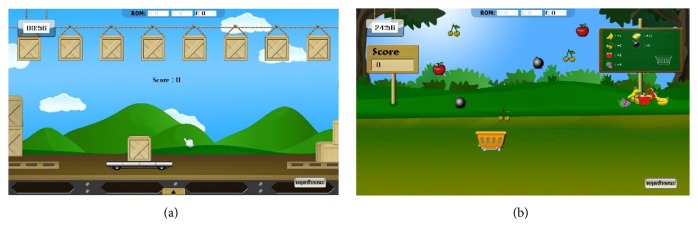
Games for Passive mode.

To play the transportation game in Figure 6(a), the patient must slide the pushcart to the left side to pick up the freight. Then the pushcart must be moved to the other side to deliver the freight. After reaching the right side, the freight randomly becomes a valuable or worthless object, which indicates the score for each round. There is no reward or obstacle that can be interacted with the patient during the motion between both sides.

For the fruit collection game in Figure 6(b), each fruit has a different score. A patient should move the basket to either side to collect a piece of desired fruit and to avoid useless stuff. When matching this game for Passive mode, patients have no chance to move freely to collect any desired fruit. Therefore, it is not appropriate for Passive mode undoubtedly.

*Scoring Design for Passive Games*. To hold attraction for the patients based on the random-value objects, the high score objects should appear less frequent than the lower ones. This strategy makes the games to be more challenging and enjoyable.

Also, there should not be any negative score because this condition might discourage the patients from playing the game.

#### 2.5.2. Game Design for Pre-Active Mode

Pre-Active mode is an Active mode with an assigned path. This mode suits anyone who has enough muscle strength to motion a decayed arm along a preprogrammed path.

*Event Conditions Design of Pre-Active Games*. In Pre-Active games, the event conditions are similar to those in the Passive games. However, the main difference is an additional condition of acting force. To do the exercise in Pre-Active mode, the patient has to exert an amount of force to the system continuously (Section 2.2). So we can create an incentive for this mode. For example, an extra reward appeared near the end just only a few seconds before the key object reaches the point. This bait stimulates the patient to continuously make an effort to play with the system.

**Figure 7:**
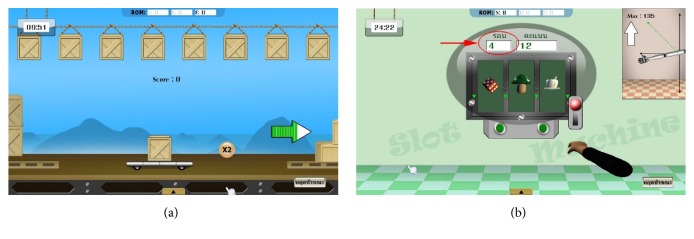
Games for Active mode.

Pre-Active Transportation game is derived from a game in Passive mode. Due to the fact that patient may not notice the magnitude of acting force, it is necessary to add a force indicator in every Active game. The indicators, which represent the force magnitudes in colors, are shown as arrows at both sides of the window in Figure 7(a).

*Scoring Design for Pre-Active Games*. Managing the score in Pre-Active games is almost identical to those games in Passive mode. Anyhow, the random-score method cannot be analyzed directly for verifying the performance of the player in the Pre-Active mode. Therefore scoring based on a number of movement cycles is implemented to evaluate the performance of each patient. The patient who has more power must be able to exercise more cycles. This can be the result of higher score. Thus the final score can imply how much effort the patient has done for each session.

For the Slot Machine Game in Figure 7(b), a number of movement cycles, which are shown in a box with a red circle, are used for calculating the score and analyzing the performance at the end of the game.

## 3. Preclinical Trial

To verify the advantages of SEFRE, we have carried out an intensive clinical trial at the national rehabilitation center of Thailand.

### 3.1. Protocol

The main objective of the preclinical trial is to validate the operation and the safety of the system through the rehabilitation in Passive mode. Since a small group of subjects can give a preliminary result to forward the work [23–25]. Thus, the three subjects who aged 40–68 years were recruited to proceed with the following trial steps:

- Before they began the trial and signed the contract, the details of the trial protocol were precisely explained. Then the personal information and medical background were recorded.
- During the 5-day trial, the subjects received the conventional therapy for two hours per session, one session per day. In each session, they must be rehabilitated by SEFRE for 15 minutes.
- In this trial, every subject was verified with two assessments, namely, the muscle tone assessment and Passive ROM (PROM) assessment. Both assessments were carried out two times: before the first day and after the fifth day.

**Figure 8:**
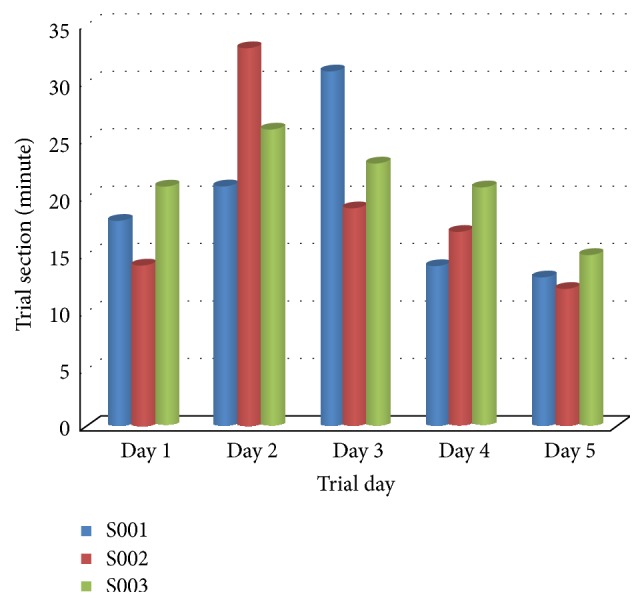
Trial period of all subjects on each day.

Also, the subjects must fill in the questionnaire to evaluate the impression of SEFRE Rehab System. The actual trial period of all subjects for each day is shown in Figure 8.

### 3.2. Movements

**Figure 9:**
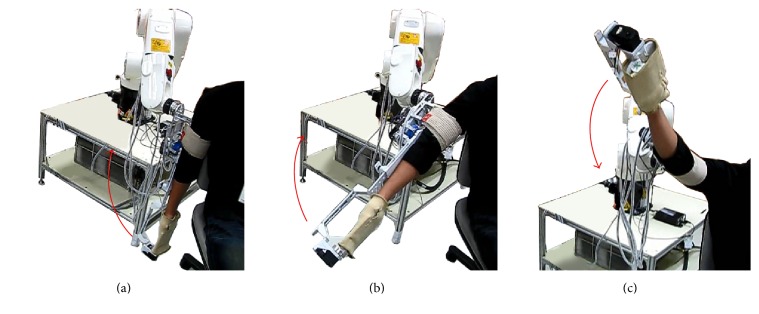
Example movements of SEFRE Rehab System: shoulder extension-flexion.

**Figure 10 fig10:**
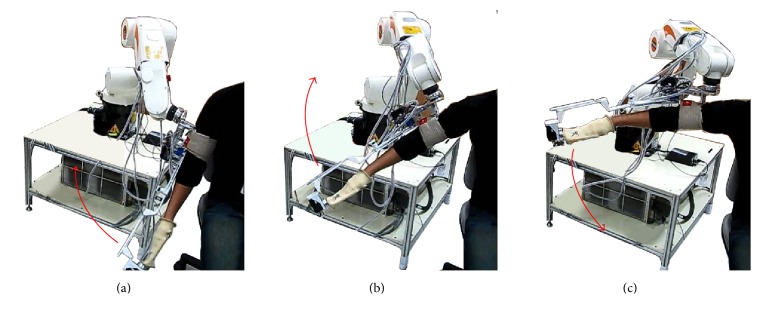
Example movements of SEFRE Rehab System: shoulder abduction-adduction.

**Figure 11 fig11:**
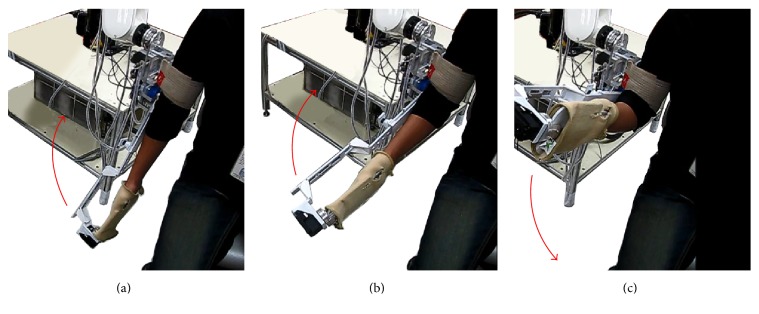
Example movements of SEFRE Rehab System: elbow extension-flexion.

**Figure 12:**
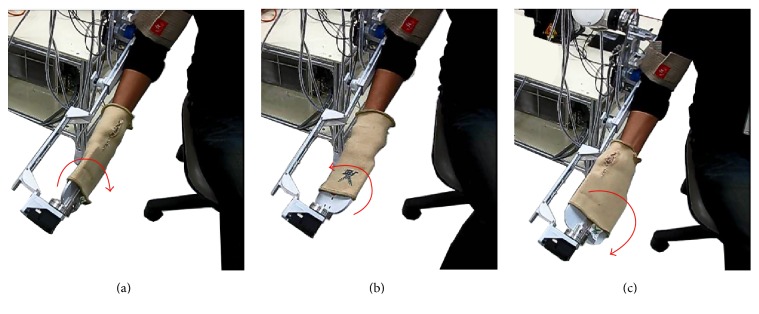
Example movements of SEFRE Rehab System: forearm pronation-supination.

SEFRE provides the exercise for the patients in several movements as shown in Figures 9 [Fig fig10] [Fig fig11]–12. Nevertheless, to optimize the trial process, three activities were set as verifying motions: shoulder flexion-extension, elbow flexion-extension, and forearm pronation-supination.

### 3.3. Trial Result

**Figure 13:**
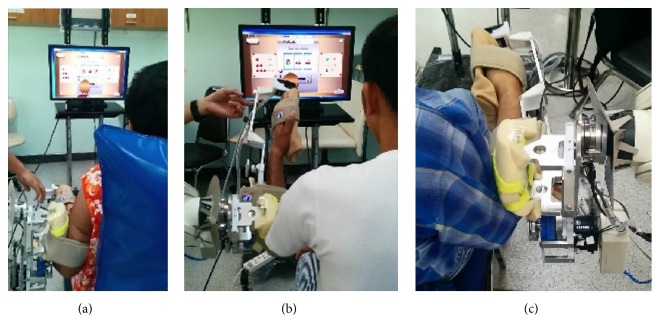
Examples of SEFRE clinical trial.

Examples of subjects for the preclinical trial are shown in Figure 13. Based on the results of our intensive trial, we believe that the benefits of SEFRE have been verified. First of all, all subjects and relatives felt safe when they were rehabilitated by SEFRE. Also, results of the muscle tone and PROM assessments were evidences that SEFRE can retain the physical condition of the upper limb (Tables 2 and 3). Furthermore, the system allows the caretaker to spend the precious time on other patients without full attention to SEFRE.

Based on these results, the next phase is to certify the system in Active mode. Also, the number of subjects and the trial period must be extended. Moreover, to assure that the system can support and unite with the conventional therapy protocol, the subjects must be divided into two groups, that is, the control and experiment groups. Thus, the work we present here is still in an initiating step; nevertheless, we believe that our research must be a great achievement to rehabilitation domain when SEFRE is accomplished.

## 4. Conclusion

SEFRE Rehab System is composed of a robotic manipulator and an exoskeleton, that is, FSM (Forearm Supportive Mechanism). The main controller of the system is the Master PC that consists of five modules, that is, Intelligent Control (IC), Patient Communication (PC), Training with Game (TG), Progress Monitoring (PM), and Patient Supervision (PS). Based on these modules, SEFRE Rehab System is able to provide six arm therapy modes: Passive (P), Passive Stretching (PS), Passive Guiding (PG), Initiating Active (IA), Active Assisted (AA), and Active Resisted (AR). These allow SEFRE to be the robotic rehabilitation system for everybody, for example, a patient without any residue muscle strength or a healthy person who has temporary muscle deficiency problem. To validate the advantages of the system, the preclinical trial was carried out by providing the rehabilitation in Passive mode for three subjects who aged 40–68 years. The results of this intensive trial, that is, three subjects were trialed for five sessions, show that all subjects and relatives felt safe when they were rehabilitated by SEFRE. Moreover, the muscle tone and PROM assessments verified the system for retaining the physical condition of the upper limb. Thus the next phase is to validate the system in Active mode, which we believe that this must be a great benefit to the rehabilitation field when the system is completed.

Furthermore, to achieve the motto of SEFRE, an affordable robotics rehabilitation system, a small industrial robot with ATI mini45 module, must be replaced with a customized novel mechanism that lower the producing cost of the system. This is our next key milestone to complete SEFRE as the Shoulder-Elbow-Forearm Robotics Economic rehabilitation system.

## Figures and Tables

**Table 1:** Examples of robotic rehabilitation.

Reference	Target	Key concept
Lum et al. [9]	Hand	A rehabilitator in bimanual lifting
Chiri et al. [10]	Hand	A novel wearable multiphalanges device
Mao and Agrawal [11]	Hand	A cable driven arm exoskeleton
Takahashi et al. [12]	Wrist	Robotic device for hand motor therapy
Krebs et al. [13]	Wrist	A robot for wrist rehabilitation
Zhang et al. [14]	Elbow	A curved pneumatic muscle based exoskeleton
Wiegand et al. [15]	Elbow	A lightweight, portable, and active orthosis
O'Malley et al. [16]	Wrist & forearm	An exoskeleton rehabilitation robot
Oblak et al. [17]	Wrist & forearm	A universal haptic drive (UHD)
Hesse et al. [18]	Elbow & wrist	A robotic arm for bilateral training
Perry et al. [19]	Upper limb	A cable-actuated dexterous powered arm exoskeleton
Howard et al. [20]	Upper limb	A modular 2D planar manipulandum
Lam et al. [21]	Upper limb	A haptic device with postural sensors

**Table 2:** SEFRE clinical trial result of 3 subjects: PROM.

Day	Shoulder E-F	Elbow E-F	Forearm P-S
1st	WNL	WNL	WNL
5th	WNL	WNL	WNL

**Table 3:** SEFRE clinical trial result of 3 subjects: muscle tone.

Day	S001	S002	S002
1st	1	1	1
5th	1	1	1
